# Insertion-trigger residues differentially modulate endosomal escape by cytotoxic necrotizing factor toxins

**DOI:** 10.1016/j.jbc.2021.101347

**Published:** 2021-10-27

**Authors:** Elizabeth E. Haywood, Nicholas B. Handy, James W. Lopez, Mengfei Ho, Brenda A. Wilson

**Affiliations:** Department of Microbiology, University of Illinois at Urbana-Champaign, Urbana, Illinois, USA

**Keywords:** bacterial toxin, drug delivery system, fusion protein, protein chimera, protein engineering, molecular evolution, small GTPase, protein deamidation, structure–function, protein translocation, ABMA, 1-adamantyl (5-bromo-2-methoxybenzyl) amine, BTIDD, bacterial toxin-inspired drug delivery, CNF, cytotoxic necrotizing factor, DMEM, Dulbecco’s modified eagle medium, DT, diphtheria toxin, EGA, 4-bromo-benzaldehyde N-(2,6-dimethylphenyl) semi-carbazone, HLH, helix-loop-helix, LPR, laminin precursor receptor, nls, nonlinear least-squares, RLU, relative light unit

## Abstract

The cellular specificity, potency, and modular nature of bacterial protein toxins enable their application for targeted cytosolic delivery of therapeutic cargo. Efficient endosomal escape is a critical step in the design of bacterial toxin-inspired drug delivery (BTIDD) vehicles to avoid lysosomal degradation and promote optimal cargo delivery. The cytotoxic necrotizing factor (CNF) family of modular toxins represents a useful model for investigating cargo-delivery mechanisms due to the availability of many homologs with high sequence identity, their flexibility in swapping domains, and their differential activity profiles. Previously, we found that CNFy is more sensitive to endosomal acidification inhibitors than CNF1 and CNF2. Here, we report that CNF3 is even less sensitive than CNF1/2. We identified two amino acid residues within the putative translocation domain (E374 and E412 in CNFy, Q373 and S411 in CNF3) that differentiate between these two toxins. Swapping these corresponding residues in each toxin changed the sensitivity to endosomal acidification and efficiency of cargo-delivery to be more similar to the other toxin. Results suggested that trafficking to the more acidic late endosome is required for cargo delivery by CNFy but not CNF3. This model was supported by results from toxin treatment of cells in the presence of NH_4_Cl, which blocks endosomal acidification, and of small-molecule inhibitors EGA, which blocks trafficking to late endosomes, and ABMA, which blocks endosomal escape and trafficking to the lysosomal degradative pathway. These findings suggest that it is possible to fine-tune endosomal escape and cytosolic cargo delivery efficiency in designing BTIDD platforms.

Modular bacterial toxins deliver their catalytic cargo into the cytosol of specific target cells. After binding and cellular uptake, these toxins transport their toxic cargo to the cytosol through multiple trafficking pathways, the most common of which involve retrograde transport through the endoplasmic reticulum or endocytic trafficking from early to late endosomes followed by pH-dependent endosomal escape. Already, a number of modular toxins have been exploited for their ability to deliver heterologous cargo molecules to the cytosol, including fluorescent proteins (1), epitope tags (2), nanobodies (3, 4), various recombinant enzymes (5, 6, 7, 8, 9, 10), and nucleic-acid-binding proteins (11, 12, 13). Bacterial toxin-inspired drug delivery (BTIDD) platforms, such as those described for the cytotoxic necrotizing factor (CNF) toxins (14) that assemble from modular components, could be expanded to noncognate therapeutic cargos if the determinants for efficient cytosolic delivery of the biologic cargo were more fully understood.

CNF toxins are Rho-deamidating toxins that access their cytosolic targets through trafficking to and escape from acidified endosomes (15). The CNF toxin family is comprised of at least nine full-length homologs sharing 54 to 84% identity (14), with the highest identity being shared by CNF1 and CNF2 at 84%. The high sequence identities, yet distinct cellular activities, observed among the CNF toxin family members enable probing for discriminatory determinants that modulate the cargo-delivery process. For example, previous investigation of four toxins from this family (CNF1, CNF2, and CNF3 from *Escherichia coli* and CNFy from *Yersinia pseudotuberculosis*) revealed differences in cargo-delivery efficiency and compatibility of intertoxin domain assembly among these four toxins (14).

A recent crystal structure of CNFy revealed five structural domains (16). The cellular receptor-binding domain is located near the N-terminus (CNFy residues 23–134). For CNF1 and CNF2, this domain binds laminin precursor receptor (LPR) (17, 18), while the cellular receptors for the other CNF toxins have not been established. Although catalytically inactive CNFy has been shown to retard entry of CNF1 into cells, CNFy does not bind LPR, suggesting an overlapping coreceptor (19). CNF1 and CNFy reportedly have an additional binding region in the C-terminus: CNF1 residues 709 to 730 bind to Lu/BCAM adhesion molecule (20), and CNFy residues 772 to 779 bind to heparan sulfates (21). Based on previously predicted functional domain organization of the CNF family (22, 23, 24), the N-terminal membrane translocation module, comprised of domains D1 and D3 (CNFy residues 135–530), facilitates endosomal escape of the C-terminal cargo, which includes domain D4 of unknown function (CNFy residues 530–700) and the catalytic Rho-deamidase domain D5 (CNFy residues 718–1014) (16), which is also consistent with the structure of the catalytic domain of CNF1 (25).

A key step in the cellular intoxication process of CNF toxins involves a pH-dependent membrane insertion that occurs in an acidic endosome (26). Based on comparative sequence analysis that predicted a similar organization in the translocation region of CNF1 to the so-called “dagger” membrane-insertion motif (helices TH8–TH9) found in the T domain of diphtheria toxin (DT) (27, 28), a model for the pH-dependent insertion step of the cargo-delivery process was previously proposed involving a putative helix-loop-helix (HLH) region (residues 350–412 in CNF1) (24). This putative HLH of CNF1 contains four highly conserved acidic residues (D373, D379, E382, E383) in the postulated loop region that were proposed to become protonated in the acidic environment of the late endosome, thereby allowing insertion as a “dagger” into the membrane. Once on the cytosolic side of the membrane, the putative HLH would again become deprotonated, locking the HLH in place and initiating the membrane translocation process and cytosolic delivery of the cargo.

However, although these four acidic residues were confirmed to be important for cargo delivery of CNF1 (24), the crystal structure of CNFy (16) did not reveal the predicted HLH structure, and it was proposed that since the structure was determined under neutral conditions, perhaps the region changes its conformation once it is in an acidic environment. Other studies have revealed that there are differences among the CNF toxins with regard to efficiency (14) and pH dependency (29) of cargo delivery. CNF1, CNF2, and CNFy toxins have differential dose-dependent responses to inhibitors of endosomal acidification (29), such as NH_4_Cl that acts as a weak base to raise the endosomal pH and bafilomycin A1 that blocks acidification by inhibition of the vacuolar ATPase proton pump. These findings suggest that there may be other protein determinants besides the four conserved acidic residues that dictate pH sensitivity and influence CNF toxin cargo delivery efficiency.

In addition to NH_4_Cl and bafilomycin A1, two other small-molecule inhibitors of cellular trafficking pathways, 4-bromo-benzaldehyde N-(2,6-dimethylphenyl) semi-carbazone (EGA) and 1-adamantyl (5-bromo-2-methoxybenzyl) amine (ABMA), have been used to investigate intoxication mechanisms of modular protein toxins that traffic through acidified endosomes to deliver their cargo into the cytosol. EGA blocks trafficking from the early endosome to the late endosome (30, 31, 32), which prevents some toxins from reaching the lower pH compartment needed for triggering membrane insertion and translocation. In contrast, ABMA reportedly blocks intoxication independent of endosomal acidification and at a stage after acidification (33) and also inhibits trafficking from the late endosome to the lysosomal degradation pathway (34). Thus, through use of NH_4_Cl, EGA, and ABMA, it may be possible to identify key points along the endosomal pathway that may differentiate among the CNF toxins: acidification, trafficking from the early endosome to the late endosome, trafficking from the late endosome to the lysosome, and/or escape of the cargo from the endosome.

Here, we compared CNF1, CNF2, CNF3, and CNFy for their sensitivity to inhibitors of endosomal acidification and trafficking using cell-based SRE-luciferase assays, performed as previously described (14, 29). We found that among the CNF toxins, CNF3 was the most tolerant to inhibition of endosomal acidification, while CNFy was the most sensitive. To identify protein determinants that respond to changes in endosomal pH and to differentiate among the toxins, we generated and characterized a series of chimeric toxins between CNF3 and CNFy and identified the putative HLH region of the translocation domain as the region responsible for discriminating their pH sensitivities. Site-specific mutational analysis identified two acidic residues within this region responsible for mediating the differential sensitivities to NH_4_Cl. CNF3 and CNFy were also investigated for their differential sensitivity to EGA or ABMA, enabling discrimination of the exit points taken by these toxins in the intoxication pathway.

## Results

### Effects of NH_4_Cl on CNF-mediated SRE-luciferase activity

**Figure 1:**
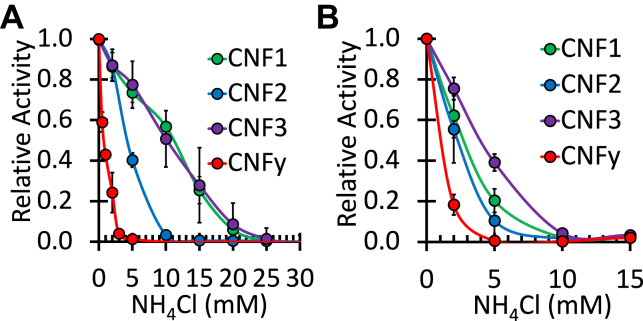
**Sensitivity of wild-type CNF toxins to endosomal acidification.** Shown are dose–response curves to NH_4_Cl treatment of the wild-type CNF toxins in the SRE-luciferase assay, as described in the Experimental procedures. HEK293T cells were treated with NH_4_Cl for 30 min prior to treatment with the indicated wild-type CNF toxins and assayed for Firefly/*Renilla* activity after 6-h incubation at a concentration of (*A*) 100 ng/ml or (*B*) corresponding to their respective EC_50_ values (0.01 nM CNF1 (*green*), 0.10 nM CNF2 (*blue*), 0.02 nM CNF3 (*purple*), and 0.80 nM CNFy (*red*)). Relative activity indicates the fold activation compared with no-inhibitor treatment. Corresponding scatter plots with all data points used to derive the best fit lines and mean values are shown in Fig. S2.

It has been established previously that agents that raise endosomal pH such as NH_4_Cl antagonize the entry of CNF toxin cargos into the cytosol (26, 29). NH_4_Cl chemically counteracts the acidification of the endosome, preventing the low pH necessary for translocation of toxin cargos. Previous studies showed that CNF1, CNF2, and CNFy had differential sensitivities to NH_4_Cl (29), while CNF3 sensitivity to NH_4_Cl was not tested previously. We first compared the activities of wild-type CNF1, CNF2, CNF3, and CNFy toxins in a cell-based SRE-luciferase assay in response to NH_4_Cl. As shown in Figure 1, pretreatment of HEK293T cells with NH_4_Cl blocked the intracellular activity of all four toxins in a dose-dependent manner. In addition to exhibiting differential cargo delivery efficiencies, as previously described (14), all four of the CNF toxins displayed differential sensitivities to inhibition of endosomal acidification at a uniform toxin dose of 100 ng/ml (Fig. 1*A*). Consistent with previously described studies for CNF1, CNF2 and CNFy (29), CNFy was the most sensitive to NH_4_Cl, CNF2 had intermediate sensitivity, and CNF1 and CNF3 were the most resistant.

As was noted previously (14), it is important to minimize the effects of differences in substrate specificities and receptor-mediated uptake when comparing CNF toxins with each other. Thus, the NH_4_Cl-sensitivity assay was also performed at toxin concentrations equivalent to their respective EC_50_ values, where the limiting step for the observed toxin activity equates to their efficiency of cargo delivery, such that differences in receptor-binding efficiencies were minimized (Fig. 1*B*). In this case, CNFy remained the most sensitive to NH_4_Cl treatment, requiring only 5 mM NH_4_Cl to block all CNFy-mediated reporter activation, whereas CNF3 remained the least sensitive. However, when tested at their respective EC_50_ values, CNF1 and CNF2 displayed similar intermediate sensitivities (Fig. 1*B*). This finding is consistent with the fact that these two toxins have the greatest sequence homology and are identical regarding charged residues in the previously identified HLH insertion region. Overall, these results suggested that CNF3 and CNFy represent the CNF toxins with the most and least sensitivity to endosomal acidification, respectively, and thus we asked whether we could identify specific protein determinants that drive this observed difference in sensitivity between them.

### Putative HLH region contains determinants mediating differential NH_4_Cl sensitivity

**Figure 2:**
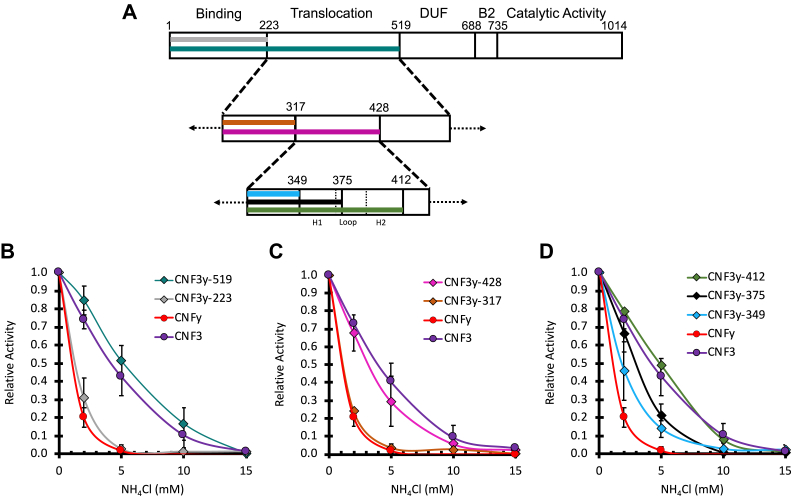
**Sensitivity of CNF3y chimeric toxins to endosomal acidification.***A*, shown is a schematic depicting the joining sites of the chimeric constructs tested in (*B*–*D*), where the functional domains of CNF3 delivery vehicle and CNFy cargo are indicated. *B*–*D*, shown are dose–response curves to NH_4_Cl treatment of the wild-type CNF3 and CNFy toxins and CNF3y chimeric toxins in the SRE-luciferase assay, as described in the Experimental procedures. HEK293T cells were treated similarly as in Figure 1*B*, where the toxin concentration used was at its EC_50_ value. Relative activity indicates the fold activation compared with no-inhibitor treatment. CNF3 (*purple circles*); CNFy (*red circles*); CNF3y-519 (*teal diamonds*); CNF3y-223 (*gray diamonds*); CNF3y-428 (*pink diamonds*); CNF3y-317 (*orange-brown diamonds*); CNF3y-412 (*green diamonds*); CNF3y-375 (*black diamonds*); CNF3y-349 (*light blue diamonds*). Corresponding scatter plots with all data points used to derive the best fit lines and mean values are shown in Fig. S3. B2, secondary binding domain; DUF4765, Domain of Unknown Function.

To identify the determinants that discriminate CNF toxin sensitivity to inhibition of endosomal acidification, we applied a binary search approach to define the region of interest (*i.e.*, the region that mediates the differential responses) and then selected new joining sites within that region to further refine the search. We generated a series of chimeric proteins with the N-terminal delivery domain of the least sensitive toxin CNF3 and the cargo domain of the most sensitive toxin CNFy (CNF3y) (Fig. 2*A*) and compared their sensitivities to NH_4_Cl inhibition in cell-based activity reporter assays, as described above. Accordingly, the chimera CNF3y-223 was joined downstream of the putative N-terminal receptor-binding domain (residues 23–134) (18, 35), while CNF3y-519 was joined upstream of the suspected cleavage site (residues 532–544) that defines the putative cargo and delivery vehicle domains (22). As shown in Figure 2*B*, CNF3y-223 was completely inhibited by 5 mM NH_4_Cl, matching the response observed for CNFy, while the inhibitor profile of CNF3y-519 resembled that of CNF3. These results confirmed that the region differentially sensing pH is located within the putative translocation domain (residues 223–519).

To minimize structural perturbations, additional joining sites at highly conserved positions 317 and 428 within the newly defined pH-sensing region were next explored, based on amino acid sequence alignment, secondary structure predictions, and regional pI calculations (data not shown). The resulting chimeric toxins CNF3y-317 and CNF3y-428, respectively, were generated and characterized using the SRE-luciferase assay. As shown in Figure 2*C*, the inhibitor profiles showed that CNF3y-317 is as sensitive to NH_4_Cl as CNFy, while CNF3y-428 is as tolerant as CNF3, thereby narrowing the pH-sensing region to positions 317 to 428, which includes the putative HLH region and the previously identified acidic residues in CNF1 (D373, D379, E382, and E383) that are important for translocation (24) and are conserved for all known CNF toxins.

To further identify the residues within the putative HLH region that contribute to the differences between CNF3 and CNFy in sensing pH changes, three new chimeric toxins were constructed: CNF3y-349, CNF3y-375, and CNF3y-412. The resulting inhibitor profiles showed that CNF3y-349 like CNFy is more sensitive to NH_4_Cl, while CNF3y-412 like CNF3 is more tolerant. Interestingly, the chimera CNF3y-375 is intermediate in sensitivity to NH_4_Cl, suggesting that one or more residues within each of the regions flanking the joining site at position 375 influence the response of the chimeric toxin to endosomal acidification. Moreover, chimera CNF3y-412 has a lower EC_50_ value of 0.056 nM, compared with CNF3y-349 and CNF3y-375, each with EC_50_ values of 0.28 nM and 0.20 nM, respectively (Table 1), further supporting the importance of this region in determining the efficiency of cargo delivery.

**Table 1:** EC_50_ values of wild-type and mutant CNF toxins^a^

Toxin	EC_50_ (nM)^b^	EC_50_ ratio^c^	
		CNF3	CNFy
CNFy	0.43 ± 0.06	—	1
CNF3y-223	0.21 ± 0.02	—	0.5
CNF3y-519	0.23 ± 0.02	—	0.5
CNF3y-428	0.052 ± 0.004	—	0.1
CNF3y-317	0.075 ± 0.006	—	0.2
CNF3y-349	0.28 ± 0.04	—	0.7
CNF3y-375	0.20 ± 0.01	—	0.5
CNF3y-412	0.056 ± 0.006	—	0.1
CNFy E374Q	0.25 ± 0.04	—	0.6
CNFy E412S	0.30 ± 0.05	—	0.7
CNFy E374Q/E412S	0.10 ± 0.09	—	0.2
CNF3	0.017 ± 0.003	1	—
CNF3 Q373E	0.045 ± 0.008	2.6	—
CNF3 S411E	0.032 ± 0.006	1.9	—
CNF3 Q373E/S411E	0.094 ± 0.019	5.5	—
CNF3 Q373K	0.13 ± 0.01	7.6	—
CNF3 E412A	0.066 ± 0.017	3.6	—
CNF3 E412K	0.095 ± 0.015	5.6	—

a The EC_50_ values were calculated from dose–response assays determined from this study.

b The EC_50_ values represented are mean ± SEM calculated using nls in R.

c The EC_50_ ratio was determined by dividing the EC_50_ of the chimeric proteins by that of the native CNF toxin with the same cargo domain A.

### Acidic residues E374 and E412 in CNFy are responsible for enhanced sensitivity to NH_4_Cl

**Figure 3:**
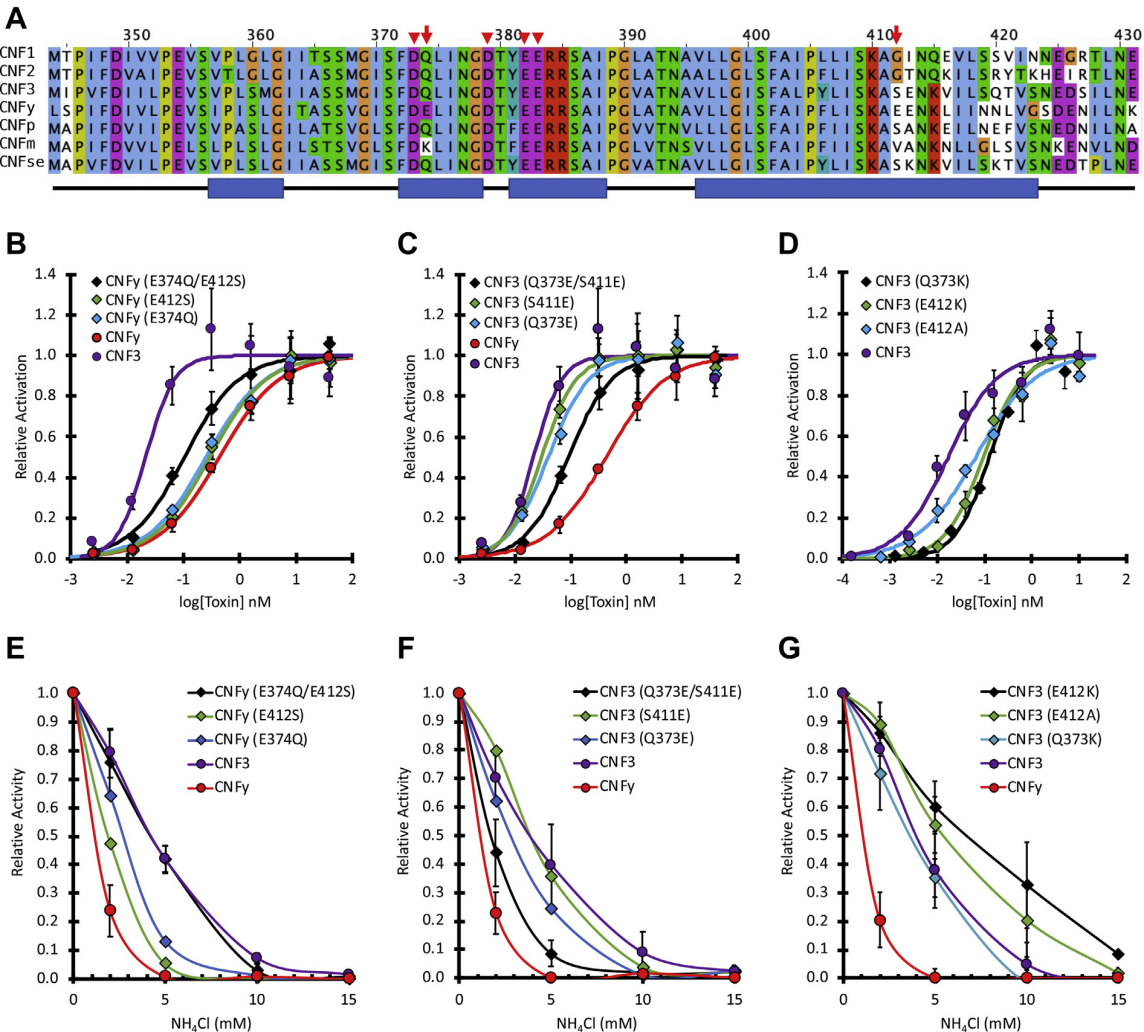
**The effect of swapping amino acid residues in the putative HLH region of CNF3 and CNFy on dose response and sensitivity to endosomal acidification.***A*, shown is the alignment of the putative HLH region of the indicated CNF toxin homologs. The scale bar shown on *top* corresponds to residue numbers for CNF1 and CNFy in this region. The four *red arrowheads* indicate the acidic residues critical for CNF1 activity. The *red arrows* indicate two critical acidic residues that distinguished between the CNF3 and CNFy sensitivity to endosomal acidification inhibitors. The *blue bars* at the *bottom* indicate the alpha helices found in the structure of CNFy (PDB 6YHK). *B*–*G*, the mutant toxins were analyzed by the SRE-luciferase assay, as described in the Experimental procedures. *B*–*D*, dose–response curves comparing point mutants of CNFy and CNF3, as indicated. *E*–*G*, effect of NH_4_Cl on the dose–response curves of mutant and wild-type CNF3 and CNFy at their respective EC_50_ concentrations, as described in Figure 1*B* and Experimental procedures. Corresponding scatter plots with all data points used to derive the best fit lines and mean values are shown in Fig. S4.

Alignment of the putative HLH insertion-trigger region in seven of the CNF homologs (CNF1, CNF2, CNF3, and CNFy, as well as CNFp from *Photobacterium damselae*, CNFm from *Moritella viscosa*, and CNFse from *Salmonella enterica*) revealed that CNFy has two additional acidic residues, E374 and E412, compared with the other CNF toxins (Fig. 3*A*), which could account for the enhanced sensitivity of CNFy to NH_4_Cl. The acidic residue E374 in CNFy was previously tested for its role in membrane translocation (19). Although the investigators found that the CNFy E374Q mutant toxin behaved like wild-type CNFy in the plasma membrane pulse experiment, they did not test the other acidic residue E412.

To further explore the roles of E374 and E412 in the differential toxin response to endosomal acidification, the amino acids at these positions in CNFy were swapped with the corresponding amino acids in CNF3 (Q373 and S411, respectively). The resulting single and double mutant toxins were examined in the cell-based SRE-luciferase assay for their relative responses (Fig. 3, *B* and *C*, respectively) and their sensitivity to NH_4_Cl inhibition (Fig. 3, *E* and *F*, respectively). The double mutant CNFy E374Q/E412S was more efficient at cargo delivery than wild-type CNFy (Fig. 3*B*) with EC_50_ values of 0.10 nM and 0.43 nM, respectively (Table 1), and more tolerant of NH_4_Cl inhibition (Fig. 3*E*), resembling the profile for CNF3.

In contrast, exchanging the corresponding residues (Q373 and S411) in the more resistant CNF3 with Glu amino acids found at the corresponding positions in CNFy generated the double mutant CNF3 Q373E/S411E that was less efficient at cargo delivery than wild-type CNF3 (Fig. 3*C*), with EC_50_ values of 0.094 nM and 0.017 nM, respectively (Table 1). The sensitivity to NH_4_Cl inhibition of this double mutant also resembled the CNFy profile (Fig. 3*F*). The corresponding single mutants of both CNFy and CNF3 exhibited intermediate response profiles.

These results are in line with the previous study mentioned above, where mutating only one of the acidic residues in CNFy was not sufficient to significantly alter its response to pH pulse (19), but by mutating both Glu residues within this region in CNFy to the corresponding Gln and Ser residues in CNF3, we were able to enhance the tolerance to acidification inhibitor, such that the double mutant CNFy E374Q/E412S escaped the endosome at a higher pH than wild-type CNFy. Thus, these two acidic residues within the putative HLH region, while not essential for cargo delivery activity, may decrease the pH required for neutralization of the structure, thereby requiring a more acidic endosome to trigger membrane insertion and cargo delivery.

### Replacing E412 in CNF3 with a nonacidic amino acid increases tolerance to NH_4_Cl

Several other CNF toxin homologs contain different charged residues at positions within the putative HLH region (Fig. 3*A*). Instead of Glu or Gln, CNFm possesses a basic Lys residue at position 374 (373 in CNF3). CNFy and CNF3 have an additional Gln residue at position 413 in CNFy (412 in CNF3), while the other CNF toxins have a neutral amino acid residue, such as Ala in CNFp and CNFm, or a basic residue such as Lys in CNFse at this position. To investigate how these residues might affect the sensitivity of the toxins to NH_4_Cl inhibition, CNF3 point mutants Q373K, E412A, and E412K were generated and examined for their responses to NH_4_Cl. The double mutant CNF3 E412A/K414Q was also generated but could not be stably expressed and tested (data not shown). Interestingly, while all three mutants were less efficient at cargo delivery than the wild-type toxin (Fig. 3*D*), the single mutants CNF3 E412A and CNF3 E412K were even more resistant to NH_4_Cl than wild-type CNF3, and CNF3 Q373K resembled wild-type CNF3 (Fig. 3*G*). These results support the importance of additional acidic residues for conferring sensitivity to endosomal acidification; however, unlike the E374Q and E412K changes in CNFy, changing E412 in CNF3 to nonacidic residues (Ala or Lys) increased acidification inhibitor tolerance but did not enhance cargo delivery efficiency.

### Differential effects of EGA and ABMA on cellular activity of CNF3 and CNFy toxins

The differential responses of CNFy and CNF3 to NH_4_Cl support a model whereby CNFy cargo requires trafficking to late endosomes to escape, while CNF3 cargo can escape from less acidic endosomes. While the acidic residues reported above accounted for the differences in sensitivity to NH_4_Cl of CNF3 and CNFy, mutations of these residues did not account entirely for the differences observed in cargo delivery efficiency. The rest of the difference could be due to a variety of other factors, including the different cargos themselves, uptake efficiencies, and differences in the trafficking pathways taken by these toxins. To further explore how CNF3 and CNFy differ in their intoxication pathways, we investigated their sensitivity to the trafficking inhibitors EGA and ABMA. Unlike NH_4_Cl, which directly affects endosomal pH, EGA treatment blocks trafficking of early endosomes to late endosomes (30, 31, 32) and so would be predicted to prevent the toxins from reaching the lower pH of the late endosomes. On the other hand, ABMA treatment does not prevent acidification, but would be predicted to accumulate the toxins in late endosomes by blocking trafficking to the lysosomal pathway (33, 34).

**Figure 4:**
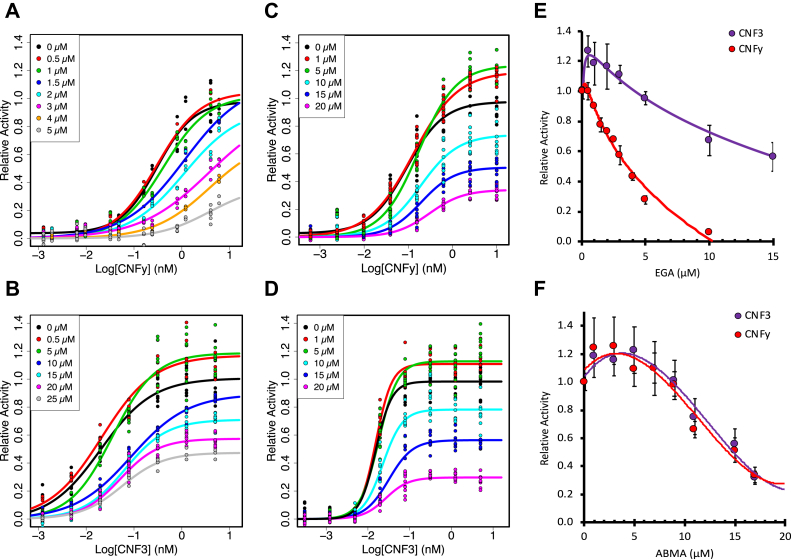
**Effect of EGA and ABMA on wild-type CNFy and CNF3 toxin activity.** HEK293T cells were treated with EGA or ABMA for 60 min prior to treatment with the indicated toxin concentration for 6 h. Cells were then lysed and analyzed by SRE-luciferase assay, as described in the Experimental procedures. *A* and *B*, shown are scatter plots for the effect of EGA on the dose–response curves for (*A*) CNFy (0 μM EGA *black*, 0.5 μM *red*, 1 μM *green*, 1.5 μM *dark blue*, 2 μM *light blue*, 3 μM *pink*, 4 μM *orange*, 5 μM *gray*) and (*B*) CNF3 (0 μM EGA *black*, 0.5 μM *red*, 5 μM *green*, 10 μM *dark blue*, 15 μM *light blue*, 20 μM *pink*, 25 μM *gray*). *C* and *D*, shown are scatter plots for the effect of ABMA on the dose–response curves for (*C*) CNFy (0 μM ABMA *black*, 1 μM *red*, 5 μM *green*, 10 μM *light blue*, 15 μM *dark blue*, 20 μM *pink*) and (*D*) CNF3 (0 μM ABMA *black*, 1 μM *red*, 5 μM *green*, 10 μM *light blue*, 15 μM *dark blue*, 20 μM *pink*). *E*, shown are the effects of EGA treatment on the activity of CNF3 toxin (*purple*) and CNFy (*red*) at toxin concentrations of 0.3 nM and 6 nM, respectively. *F*, shown are the effects of ABMA treatment on the activity of CNF3 toxin (*purple*) and CNFy (*red*) at toxin concentrations of 0.25 nM and 10 nM, respectively. Concentrations of inhibitor higher than 40 μM for ABMA and 50 μM for EGA were toxic to the cells (data not shown). Corresponding scatter plots with all data points used to derive the best fit lines and mean values are shown in Fig. S5.

With this in mind, we first investigated wild-type CNFy and CNF3 toxins for their sensitivity to EGA. HEK293T cells were pretreated with EGA for 1 h and then incubated with wild-type toxin at varying concentrations for 6 h. As shown in Figure 4*A*, CNFy was inhibited in a dose-dependent manner by EGA at concentrations higher than 1 μM. In contrast, as shown in Figure 4*B*, CNF3 was only inhibited at EGA concentrations of 10 μM or higher. This is consistent with their responses to NH_4_Cl treatment (see Fig. 1). Interestingly, unlike CNFy, CNF3 showed enhanced activity at EGA concentrations of 5 μM or lower. A similar enhancement was previously noted for CNF1 and CNF2 with other endosomal acidification inhibitors, such as NH_4_Cl and bafilomycin A1 (29). This indicates that EGA is inhibiting these toxins at a step that differentiates their cargo-delivery pathways by preventing CNFy from reaching more acidic environments required for delivery of its cargo. Like CNF1, CNF3 can deliver its cargo at a higher pH, and therefore, it is less inhibited by EGA than CNFy at all EGA concentrations (see Fig. 1).

Unlike EGA, which displayed different effects on the dose–response curves of CNFy and CNF3, ABMA had the same effect on the activities of both toxins, with both toxins displaying similar dose–response curves. At lower ABMA concentrations of 5 μM and 1 μM, cellular activity of CNFy and CNF3 was equally enhanced, while at higher ABMA concentrations, both toxins were inhibited with similar dose–response profiles (Fig. 4, *C* and *D*, respectively). This suggests that ABMA acts at a common step in the intoxication pathways of these toxins, presumably by blocking their cargos from escaping the endosomes, regardless of the pH at which this occurs.

We noticed that the dose-dependent effects of EGA and ABMA were most pronounced at concentrations of CNFy and CNF3 greater than their respective EC_50_ values. So, we next explored the toxin responses using a wider range of EGA and ABMA concentrations and using higher toxin concentrations that were at ten times their respective EC_50_ values (CNF3 at 0.25 nM and CNFy at 10 nM). Consistent with the results shown in Figure 4*A*, CNFy was inhibited at all EGA concentrations tested, with complete inhibition observed at 10 μM (Fig. 4*E*). In contrast, EGA had a biphasic effect on CNF3 cargo delivery activity, enhancing CNF3 activity at EGA concentrations lower than 5 μM, but inhibiting toxin activity at higher EGA concentrations (Fig. 4*E*), albeit only partially, even at EGA concentrations up to 50 μM (data not shown). For both toxins, concentrations of ABMA lower than 9 μM enhanced toxin responses, while higher ABMA concentrations (up to 20 μM) inhibited responses of both toxins, with similar dose–response profiles (Fig. 4*F*). Concentrations of ABMA higher than 40 μM completely inhibited both toxins, but also showed significant cellular toxicity (data not shown). As ABMA acts to prevent trafficking to lysosomes, these results suggest that at low concentrations, ABMA retains the toxins in endosomes and thereby enhances cargo escape of both toxins, irrespective of the extent of acidification. However, at higher concentrations, ABMA inhibits both toxins equally regardless of the extent of acidification at which each is triggered to escape, presumably by blocking the membrane translocation process through an as-yet unknown mechanism.

## Discussion

Toxin-based cargo delivery vehicles could provide alternative platforms for efficient delivery of biologics that require access to the cytosol to reach their intracellular targets. Understanding the specific requirements and determinants of cargo delivery for these toxins is crucial for adapting them for efficient cytosolic delivery of a wide range of biologics. Here, we took advantage of the differences in pH-dependent endosomal escape among members of the full-length CNF toxin family to identify key residues responsible for optimal cargo delivery efficiency and to gain insights into determinants that sense endosomal pH and trigger escape.

Four acidic residues in the loop of the putative HLH region constitute the proposed insertion-trigger motif previously shown to be essential for cellular toxicity of CNF1 (24). In the proposed intoxication model, these residues, which are shared by all CNF toxins, are presumably deprotonated during the acidification of the endosome, triggering a conformation shift that allows insertion into the membrane and translocation, similar to what is proposed to occur with the dagger motif in DT (36). Previous studies reported differences in the sensitivities of CNF toxins to varying pH and intoxication inhibitors (19, 29). While the underlying mechanism driving these differences was not well understood, these findings strongly suggested that there are additional protein determinants that differentiate among the CNF toxins with regard to their response to endosome acidification and efficiency of cargo escape from endosomes.

Our results indicate that CNF3 is the most resistant to NH_4_Cl treatment, while CNFy is the most sensitive, and CNF1 and CNF2 are intermediate, suggesting that CNF3 escapes the endosome at a higher pH than the others, and CNFy requires more acidic late endosomes for escape. To identify the additional pH-sensitive region responsible for their differential responses to NH_4_Cl, we used a series of chimeric proteins generated by swapping varying regions in the N-terminus of the most sensitive CNFy with the same region in the least sensitive CNF3. By observing the sensitivity of the delivery of CNFy cargo to NH_4_Cl inhibition, we were able to narrow down the region conferring sensitivity to within the putative insertion-trigger motif between residues 349 and 412.

Mutating residues E373 and E412 within this region in CNFy to the corresponding uncharged residues in CNF3 not only made the mutants as resistant to NH_4_Cl as CNF3 but also enhanced their cargo-delivery efficiency, decreasing the EC_50_ value of 0.43 nM for wild-type CNFy to 0.10 nM for the CNFy double mutant. The reciprocal swapping of the nonacidic residues Q373 and S411 in CNF3 with the acidic residues from CNFy had the opposite effect on CNF3, both increasing sensitivity to NH_4_Cl and decreasing cargo-delivery efficiency as evidenced by the increased EC_50_ value from 0.017 nM for wild-type CNF3 to 0.094 nM for the CNF3 double mutant. Thus, we identified two discriminatory acidic residues in the insertion-trigger motif of CNFy, E374, and E412, which among the CNF toxins are unique to CNFy and confer a lower pH requirement for CNFy cargo delivery. Our results further suggest that neutralizing the negative charges of these two amino acid residues from the insertion-trigger motif of CNFy to resemble CNF3 promotes more efficient cargo delivery, presumably by lowering the number of total negative charges that are needed to be shielded for membrane insertion to occur. This then allows the toxin cargo to escape at a higher pH and avoid lysosomal degradation.

Since CNF3 is the most sensitive to endosomal acidification, escaping at a higher endosomal pH, we next asked whether replacing additional acidic residues in CNF3 would increase its resistance to NH_4_Cl and improve its efficiency of cargo delivery. Our results showed that replacing E412 in CNF3 with the nonacidic amino acids Lys or Ala found in the other CNF variants CNFse or CNFm, respectively, increased its resistance to NH_4_Cl over wild-type CNF3. However, unlike the similar removal of extra acidic residues in CNFy, which enhanced the cargo-delivery efficiency of CNFy, these swaps in CNF3 increased the EC_50_ values from 0.017 nM for wild-type to 0.066 nM and 0.095 nM for the mutants CNF3 E412A and E412K, respectively. These findings suggest that CNF3, unlike the other CNF toxins, may already be evolutionarily optimized to deliver its cognate cargo to the cytosol. We posit that adjusting the number of negative charges within this region in other CNF toxins that are not yet optimized for cargo-delivery efficiency may enable optimal timing of membrane insertion and subsequent endosomal escape. This also prevents trafficking to the lysosomal degradation pathway, thereby improving the activities of the toxins.

**Figure 5:**
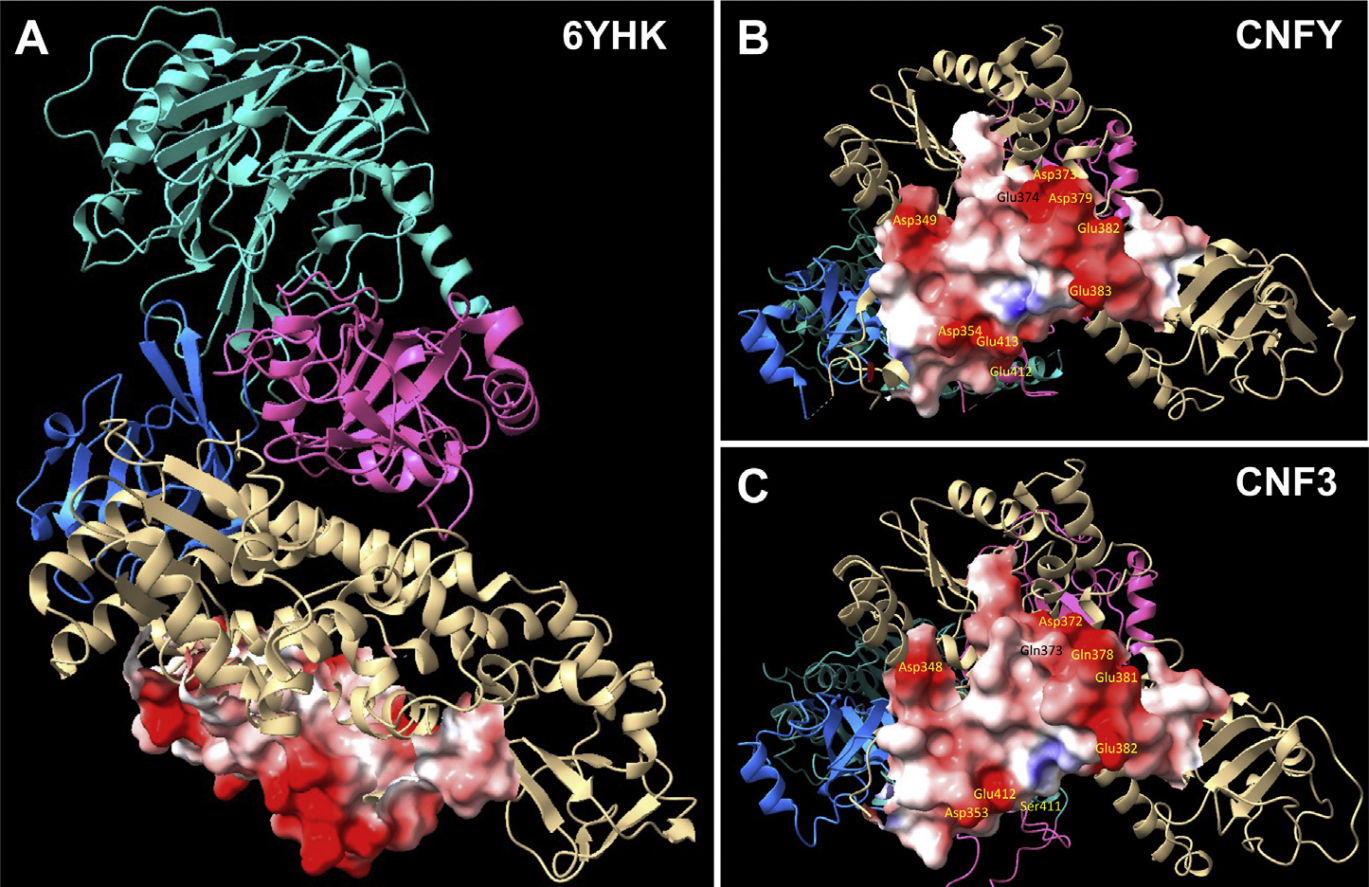
**Structures of the proposed insertion trigger regions in CNFy and CNF3.***A*, shown is a ribbon diagram representation of the structure of CNFy (PDB 6YHK) generated using ChimeraX. *Cyan*, activity domain (residues 718–1014). *Pink*, domain of unknown function (residues 522–700). *Blue*, subdomain of translocation module (residues 424–522). *Beige*, N-terminal translocation and receptor-binding module (residues 1–424). The electrostatic surface is shown for residues 344 to 423. *B*, shown is a *bottom view* of the structure in (*A*) with acidic residues labeled. *C*, shown is a similar view of the structure of CNF3 generated by HHpred-Modeller using the CNFy structure as the template, with the residues corresponding to those in (*B*) labeled.

As mentioned before, the structural topology of the putative HLH region in the crystal structure of CNFy (16) did not match its proposed role as a pH-trigger for the “dagger” membrane-insertion model (24). To explore this further in light of our identification of two additional pH-sensing acidic residues that discriminate CNFy and CNF3, we mapped the electrostatic surface for this region (residues 344–423) in the structure of CNFy. As illustrated in Figure 5, *A* and *B*, nine acidic residues define two parallel ridges, (D349, D354, E412, and E413) and (D373, E374, D379, E382, and E383), along one surface of the toxin protein that comprises the delivery vehicle module. The previously identified pH-sensing residues (D373, D379, E382, and E383) are localized along the second ridge. CNFy possesses an additional pH-sensing acidic residue E374 long this ridge, while the other pH-sensing acidic residue E412 lies along the other ridge. These two acidic residues are absent in the other CNF toxins. As illustrated in the electrostatic surface model of CNF3 (Fig. 5*C*), generated by homology modeling using the CNFy structure as a template, the corresponding residues in CNF3 (Q373 and S411) reduce the negative charges of the surface. These results suggest an alternative mechanism for membrane–protein surface interaction and insertion upon neutralization of these acidic charges in response to pH changes in the endosome.

To further investigate the different pathways CNFy and CNF3 take to deliver their cargo, we tested them for sensitivity to the trafficking inhibitors EGA and ABMA. At lower concentrations of EGA, CNF3 activity was enhanced while CNFy showed no enhancement. CNF3 can escape from the early endosomes and is prevented from entering the degradation pathway, while CNFy is unable to reach the low pH it needs to escape. For the same reason, higher EGA concentrations inhibited the activity of CNFy more than that of CNF3. Complete inhibition of CNF3 activity at a toxin concentration of 0.3 nM was not observed at EGA concentrations up to 50 μM. In contrast, complete inhibition of CNFy activity even at a toxin concentration of 6 nM was achieved at EGA concentrations greater than 10 μM. These findings support the model that even in the presence of relatively high EGA concentrations, CNF3 does not require trafficking to late endosomes, while CNFy does, and that early endosomes are acidified sufficiently such that a portion of CNF3 cargo is still able to escape.

In contrast to EGA, low ABMA concentrations equally enhanced the activity of CNF3 and CNFy, presumably by blocking trafficking to the lysosomal pathway. At higher concentrations of ABMA, both CNF3 and CNFy were again equally inhibited, presumably by ABMA blocking their escape from the acidified endosomes. Since CNF3 escapes from early endosomes and CNFy from late endosomes, indiscriminate blocking of CNF3 and CNFy by ABMA suggests that ABMA inhibits escape from both early and late endosomes, presumably after the pH-sensitive membrane insertion step.

**Figure 6:**
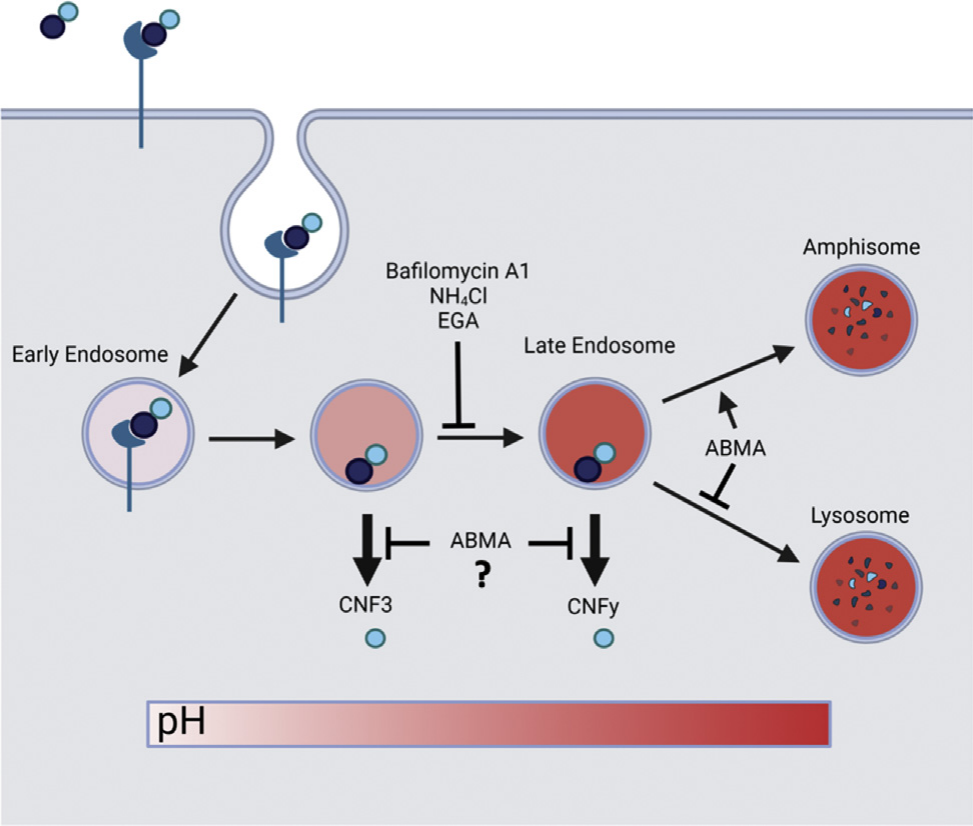
**Proposed mechanism of cytosolic cargo delivery by CNF3 and CNFy toxins.** Shown is a diagram of a proposed mechanism for intoxication and cytosolic cargo delivery by CNF3 and CNFy. Based on results from their differential response to NH_4_Cl treatment, endosomal escape of CNF3 cargo is proposed to occur at a higher endosomal pH than for CNFy cargo. At high concentrations of inhibitor, both EGA and ABMA blocked CNF3 and CNFy activity, with EGA inhibiting CNFy more than CNF3 and ABMA blocking both toxins equally. However, at low inhibitor concentrations, CNF3 and CNFy toxin activities were enhanced by both inhibitors, presumably due to the inhibitors preventing trafficking to the lysosomal degradative pathway, which in each case enabled more toxin to escape from the endosome. Since CNF3 can escape the endosome at a higher pH than CNFy, EGA blockade of early to late endosomal trafficking enhanced CNF3 activity more than CNFy activity.

Based on these results, we propose the pathways of toxin trafficking and cargo delivery illustrated in Figure 6, where CNF3 leaves the endosome at a point earlier than CNFy and is more resistant to inhibition by NH_4_Cl and EGA, because unlike CNFy, CNF3 does not need the more acidic environment of the late endosome for cargo escape. At high concentrations of ABMA, both toxins were inhibited equally, regardless of the pH that triggers their membrane insertion. In each case, low concentrations of EGA and ABMA prevented toxin trafficking to lysosomes and thereby enhanced endosomal escape of the cargo.

As modular protein toxins are increasingly employed as potential cytosolic delivery platforms for therapeutics (8, 37, 38, 39), it is imperative that we continue to investigate the intricate regulation of their cytosolic cargo-delivery mechanisms to optimize their therapeutic potential. We anticipate results from these studies will inform our understanding of the pH-dependent translocation mechanism of CNF toxins and will have broader implications for the development of BTIDD platforms.

## Experimental procedures

### Amino acid alignment of CNF toxin homologs

Sequences for comparisons were obtained from NCBI: Accession # CAA50007 CNF1, Accession # WP057108870 CNF2, Accession # WP024231387 CNF3, Accession # WP012304286 CNFy, Accession # WP045110427 CNFm, Accession # WP005306733 CNFp, Accession # WP079952502 CNFse.

The alignment was generated using MUSCLE (40) and visualized using Jalview (41) to color the amino acid residue sequences in Clustal format.

### Construction and purification of CNF toxin constructs

Plasmids encoding the genes for CNF1 (pQE-CNF1), CNF2 (pProEx-CNF2), and CNFy (pQE-CNFy) were obtained as previously described (29). CNF3 was constructed utilizing IDT gBlock DNA fragments designed based on GenBank # AM263062.1. The CNF1, CNF2, CNF3 and CNFy genes were recloned into the pSuperG vector as previously described (14). To generate chimeric toxins, restriction enzyme sites were introduced into the CNF gene sequences corresponding to amino acid position 223 in the CNF3 and CNFy proteins with conservative mutations. Joining at amino acid 519 was carried out by inserting a restriction site that resulted in the three amino acid insertion, CNF3 F514 – YVS – CNFy G519. The joining sites at positions 317, 349, 375, 412, and 428 were generated using overlapping PCR primers, as were the single and double amino acid mutations.

The His_6_-tagged CNF proteins were expressed in *E. coli* Top10 cells and partially purified as previously described (14). Briefly, the cells were harvested by centrifugation, resuspended in lysis buffer, and lysed by sonication, followed by high-speed centrifugation to isolate the soluble fraction, which was then purified by affinity chromatography using a Ni^2+^-NTA-agarose column (Qiagen) and anion-exchange chromatography using a HiTrapQ column (GE Healthcare Life Sciences). The resulting pooled protein fractions were desalted by gel filtration chromatography using a PD-10 column (GE Healthcare Life Science), eluting with PBS containing 10% glycerol. All proteins were quantified by NIH ImageJ digital image analysis of Coomassie-stained SDS-PAGE gels using BSA as the standard. Summary of SDS-PAGE gels of the toxin samples used in the assays is shown in Fig. S1. Toxin samples were flash frozen and stored at −80 °C until use.

### Cell culture

HEK293T cells (ATCC # CRL-11268) were cultured in Dulbecco’s Modified Eagle Medium (DMEM, Gibco-Invitrogen), supplemented with 0.37% sodium bicarbonate, 100 U/ml penicillin-streptomycin (Thermo Fisher Scientific), and fetal bovine serum (HyClone FBS, Thermo Fisher Scientific). Cells were maintained in DMEM with 5% FBS.

### SRE-luciferase assays

HEK293T cells in 24-well plates at 80% confluency were transfected using the calcium phosphate method, as previously described (29). Briefly, culture medium was changed to 2% FBS DMEM immediately prior to transfection. Cells were transfected with two plasmids, one containing an SRE promoter fused to a firefly luciferase reporter gene (pSRE-luc, Stratagene) and the other containing an HSV-TK promoter fused to a *Renilla* luciferase gene, which served as a low-expression constitutive reporter control gene (pGL4.74 hRluc/TK, Promega) at a final DNA concentration in each dish of 3.2 μg/ml pSRE-luc and 0.1 μg/ml pGL4.74 hRluc/TK. While vortexing, a solution of the plasmids in 250 mM CaCl_2_ was added dropwise to a solution of 2× HEPES-buffered saline, and the resulting solution was incubated at room temperature for 20 min and then added dropwise to each dish. Cells were incubated for 7 h at 37 °C and 5.5% CO_2_ and then split 1:1 into a 24-well or 48-well plate and further incubated for 16 to 18 h. For inhibitor experiments, fresh DMEM containing NH_4_Cl, ABMA, or EGA was then added to the wells to give the indicated final concentrations and further incubated for 30 min for NH_4_Cl or 60 min for ABMA and EGA. DMEM containing toxin was added to the indicated final toxin concentration, and the cells were further incubated. After 6 h, the medium was removed and the cells were lysed with 100 μl per well of Passive Lysis Buffer (Promega). After 15 min incubation on a rocker, 25 μl of sample from each well was transferred to a 96-well plate well, and the lysates were analyzed for firefly luciferase reporter activity and the constitutive *Renilla* luciferase control activity using the Promega Dual-Luciferase Reporter Assay System by addition of 25 μl of Luciferase Assay Reagent, followed by 25 μl of Stop and Glo Buffer per well, according to manufacturer's protocol. Luminescence was measured using a Synergy-HT multidetection microplate reader (BioTek), and results were generated using the Biotek microplate software Gen5 and reported as relative light units (RLUs), with settings: sensitivity = 108 and integration time = 1 s. Experiments were performed at least three independent times in triplicate.

### Data analysis

SRE-luciferase activity was determined by dividing the firefly RLUs by the *Renilla* control RLUs. The fold activation was corrected by subtracting the mean SRE-luciferase activity for the untreated samples from the toxin-treated samples. The fold activation for each well was compiled and analyzed with the nonlinear least-squares (nls) function in RStudio (URL: http://www.rstudio.com; URL: https://www.R-project.org) to create a best fit, three-parameter logistic (3PL) equation:

$$y=F\left(x\right)=\frac{A}{1+{\left(\frac{X}{C}\right)}^{B}}$$

where *A* = maximum asymptote, *B* = slope, and *C* = point of inflection or the EC_50_ value. The fold activation was divided by the calculated maximum to normalize the curves to a maximum of 1. The normalized fold activation was then analyzed *via* the same nls() equation. The standard error for the EC_50_ values was calculated using nls() in R. Each data point represents the mean of at least three independent experiments performed in triplicate. Data represented are the mean ± standard error of the mean (SEM). For the inhibitor response curves, the fold activation of control PBS-treated cells was subtracted from each data point and then normalized to the no-inhibitor treatment for that toxin.

### Homology modeling

The HH suite website (URL: https://toolkit.tuebingen.mpg.de/tools/hhpred) was used for homology modeling (42). Alignment of CNF3 and other CNF homologs was used as query for the HHpred search. The hit for CNFy, PDB 6YHK, was selected as a template for generating a 3D structure using MODELLER (43) on the HH suite website. The resulting structure was used as a CNF3 model. The UCSF ChimeraX program (44) was used to visualize both the CNFy structure (PDB 6YHK) and the CNF3 model.

## Data availability

All data described in the manuscript are contained within the manuscript and supporting information or available through publicly assessable repositories at the indicated URLs.

## Supporting information

This article contains supporting information.

## Conflicts of interest

The authors declare that they have no conflicts of interest with the contents of this article.
