# Supplementary material for: Insertion-trigger residues differentially modulate endosomal escape by cytotoxic necrotizing factor toxins
Source: J Biol Chem. 2021 Oct 27;297(5):101347. doi: 10.1016/j.jbc.2021.101347 (PMC8592880; doi:10.1016/j.jbc.2021.101347)
Supplement: Supplemental Figures S1–S5 [file mmc1.pdf]

Insertion-trigger residues differentially modulate cargo delivery by cytotoxic necrotizing factor toxins

Elizabeth E. Haywood[1#], Nicholas B. Handy[1], James W. Lopez[1], Mengfei Ho[1], and Brenda A. Wilson[1*]

[1]From the Department of Microbiology, University of Illinois at Urbana-Champaign, Urbana, IL

[#]Current address: Department of Biology, Illinois Wesleyan University, Bloomington, IL

* To whom correspondence should be addressed: Dr. Brenda A. Wilson, Department of Microbiology, University of Illinois Urbana-Champaign, 601 S. Goodwin Ave Urbana, IL 61801,
telephone: (217) 244- 9631; fax: (217) 244-6697; E-mail: wilson7@illinois.edu

Running title: *Insertion-trigger residues modulate cargo delivery*

Keywords: bacterial toxin, drug delivery system, fusion protein, protein chimera, protein engineering, molecular evolution, small GTPase, protein deamidation, structure-function, protein translocation

Supporting Information

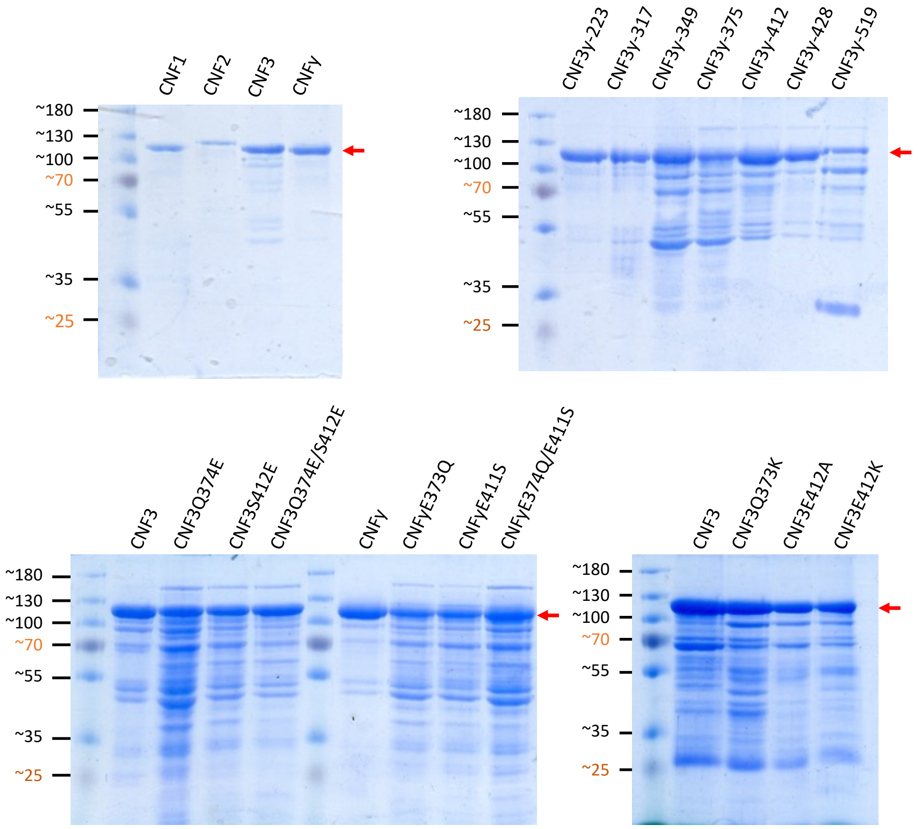

Figure S1. SDS-PAGE gels of toxins used in assays. Shown are the partially purified proteins (~4 µg per lane) run on 10% SDS-PAGE gels stained with Coomassie Brilliant Blue. Red arrow indicates expected band size for the CNF proteins.

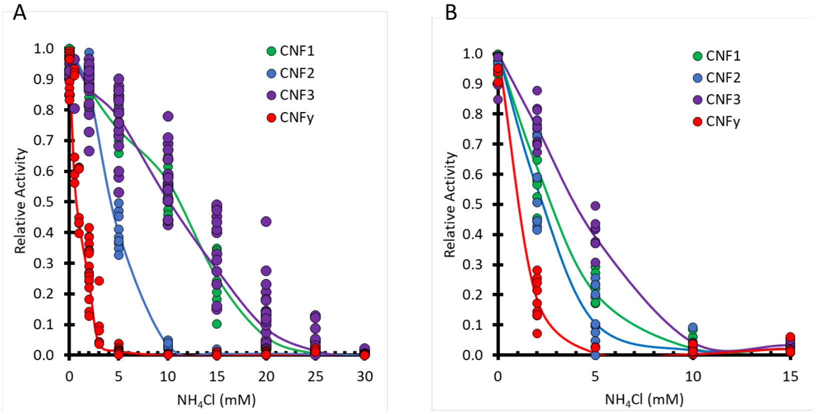

Figure S2. Sensitivity of wildtype CNF toxins to endosomal acidification. (A-B) Shown are the corresponding scatter plots with all data points used to derive the respective dose response curves in Figure 1A-1B.

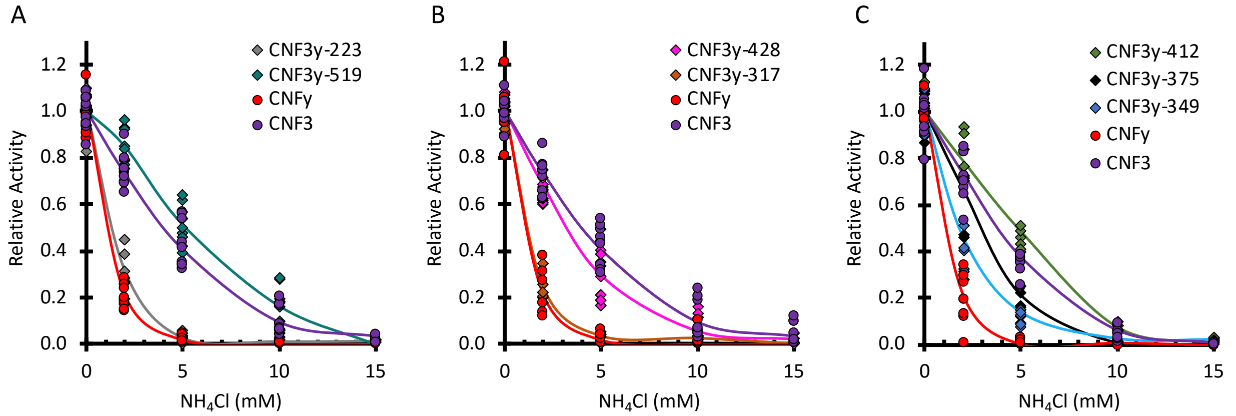

Figure S3. Sensitivity of CNF3y chimeric toxins endosomal acidification. (A-C) Shown are the corresponding scatter plots with all data points used to derive the respective dose response curves in Figure 2B-2D.

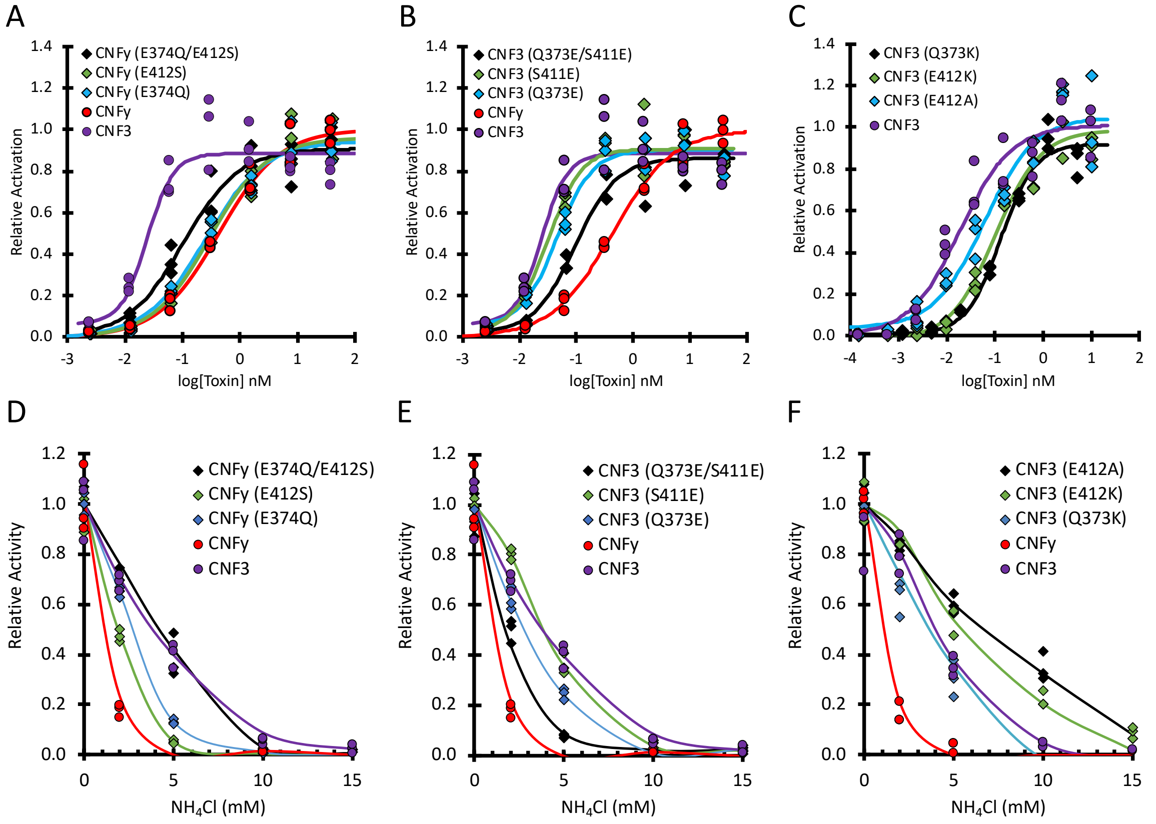

Figure S4. The effect of swapping amino acid residues in the helix-loop-helix region of CNF3 and CNFy on dose response and sensitivity to endosomal acidification. (A-F) Shown are the corresponding scatter plots with all data points used to derive the respective dose response curves in Figure 3B-3G.

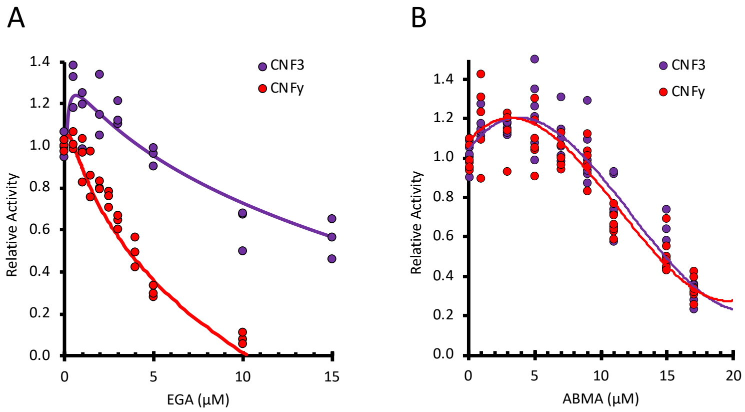

Figure S5. Effect of EGA and ABMA on wildtype CNF3 and CNFy toxin activity. (A-B) Shown are the corresponding scatter plots with all data points used to derive the respective dose response curves in Figure 5A-5B.
